# Intranasal 17β-Estradiol Modulates Spatial Learning and Memory in a Rat Model of Surgical Menopause

**DOI:** 10.3390/pharmaceutics12121225

**Published:** 2020-12-17

**Authors:** Alesia V. Prakapenka, Veronica L. Peña, Isabel Strouse, Steven Northup-Smith, Ally Schrier, Kinza Ahmed, Heather A. Bimonte-Nelson, Rachael W. Sirianni

**Affiliations:** 1 Department of Psychology, Arizona State University, Tempe, AZ 85281, USA; aprakape@asu.edu (A.V.P.); vlpena1@asu.edu (V.L.P.); istrouse@asu.edu (I.S.); snorthup@asu.edu (S.N.-S.); aschrie1@asu.edu (A.S.); kahmed9@asu.edu (K.A.); Bimonte.Nelson@asu.edu (H.A.B.-N.); 2 School of Life Sciences, Arizona State University, Tempe, AZ 85281, USA; 3 Arizona Alzheimer’s Consortium, Phoenix, AZ 85014, USA; 4 Vivian L. Smith Department of Neurosurgery, UTHealth Medical School, Houston, TX 77030, USA

**Keywords:** estrogen, intranasal, cyclodextrin, delivery, menopause, learning, memory

## Abstract

Exogenously administered 17β-estradiol (E2) can improve spatial learning and memory, although E2 also exerts undesired effects on peripheral organs. Clinically, E2 has been solubilized in cyclodextrin for intranasal administration, which enhances brain-specific delivery. Prior work shows that the cyclodextrin structure impacts region-specific brain distribution of intranasally administered small molecules. Here, we investigated (1) cyclodextrin type-specific modulation of intranasal E2 brain distribution, and (2) cognitive and peripheral tissue effects of intranasal E2 in middle-aged ovariectomized rats. First, brain and peripheral organ distribution of intranasally administered, tritiated E2 was measured for E2 solubilized freely or in one of four cyclodextrin formulations. The E2-cyclodextrin formulation with greatest E2 uptake in cognitive brain regions versus uterine horns was then compared to free E2 on learning, memory, and uterine measures. Free E2 improved spatial reference memory, whereas E2-cyclodextrin impaired spatial working memory compared to their respective controls. Both E2 formulations increased uterine horn weights relative to controls, with E2-cyclodextrin resulting in the greatest uterine horn weight, suggesting increased uterine stimulation. Thus, intranasal administration of freely solubilized E2 is a strategic delivery tool that can yield a cognitively beneficial impact of the hormone alongside decreased peripheral effects compared to intranasal administration of cyclodextrin solubilized E2.

## 1. Introduction

Menopause is a natural aspect of aging for women, occurring at an average age of 52 years [1]. The onset of transitional or surgical menopause is accompanied by an overall decrease in circulating ovarian hormone levels. This decline in hormones is associated with undesired symptoms, including cognitive changes, vaginal atrophy, hot flashes, and osteoporosis [1,2,3]. Multiple hormone therapy options, varying by delivery system, route, and regimen, are clinically available to alleviate the presence and severity of menopause-related symptoms [4,5,6,7]. In women who have an intact uterus, estrogen-only hormone therapy is associated with an increase in the risk for developing endometrial hyperplasia and cancer; minimal uterine exposure to estrogens when administered without an opposing agent is desired [1]. Progestogens oppose estrogen-associated risks and are often administered in combination with estrogens in hormone therapy formulations [1]. However, the addition of a progestogen can also oppose estrogens’ beneficial effects (e.g., cognition, cardiovascular risk) as shown in women and animal models [2,3,8,9,10,11,12]. For example, 17β-estradiol (E2), a common estrogenic component in hormone therapy, improves spatial learning and memory in ovariectomized (Ovx) rats following subcutaneous administration, a cognitive effect that can be reversed by the addition of a progestogen [8,12,13]. Thus, increasing E2 delivery to brain regions involved in both spatial learning and memory and in E2-induced cognitive effects, specifically the frontal cortex and dorsal hippocampus [12,14,15], while bypassing peripheral tissues, could negate the clinical requirement for an opposing progestogen and maximize the therapeutic potential of E2.

The intranasal route of administration circumvents first-pass metabolism and can facilitate specific accumulation of agents in brain versus peripheral tissue compared to other administration routes, including subcutaneous, oral, and intravenous [16,17,18,19,20]. Several clinical trials have evaluated the efficacy of intranasal administration of multiple types of hormones (e.g., oxytocin, testosterone, E2, gonadotropins) to treat vasomotor symptoms, infertility, voice quality, and other target symptoms [21,22,23,24,25,26]. Indeed, intranasal E2 is a promising hormone delivery system that can produce beneficial effects to combat menopause-related symptoms, similar to the effects of oral and transdermal hormone delivery systems, and can offer additional advantages by minimizing peripheral E2 burden, such as lower breast density with intranasal versus oral E2 [27,28,29]. An intranasal E2 spray, Aerodiol, was previously clinically available for menopausal hormone therapy with no severe side effects reported, although this specific formulation was later discontinued [16,30]. Clinical evaluations concluded that a daily administration of Aerodiol was well tolerated and effective at reducing symptoms associated with menopause in women [16,30], which highlights the significance of this mode of delivery for treatment of symptoms following depletion of endogenous E2. Intranasal administration of E2 yields cyclic E2 circulating levels, a hormone profile that more closely resembles the natural fluctuations in endogenously circulating E2 levels in both women and animal models [16,17]. Furthermore, in healthy postmenopausal women, a single dose of intranasally administered E2 increases cerebral and cerebellar blood flow but not peripheral blood flow [31,32], suggesting an important role for the intranasal route of E2 administration in regulating brain specific effects while minimizing peripheral effects of E2.

A major limitation in administering E2 via the intranasal route is the hormone’s low aqueous solubility. In Aerodiol, E2 was solubilized with the addition of randomly methylated β-cyclodextrin, which is a member of the cyclodextrin (CD) family of cyclic oligosaccharides. CDs are composed of glucose molecules bound together in a ring, forming a hydrophobic core with a hydrophilic surface. CDs are commonly used in drug delivery to solubilize hydrophobic compounds by creating an inclusion complex. CD structures differ based on the number of glucose chains (6-, 7-, and 8- chains), and these structural properties can be exploited to optimize solubilization and tissue distribution of small molecule-CD complexes. A number of CD formulations are available commercially. For instance, 2-hydroxypropyl β-CD (2HP-β-CD) is used to produce a commercially available “water-soluble E2” (Sigma-Aldrich, St. Louis, MO, USA). Of note, this E2-CD complex has been previously utilized in behavioral rodent paradigms to assess the role of E2 in cognitive function [33,34,35].

The question of how CD solubilization impacts E2 distribution and activity has not yet been directly addressed. Rodent studies suggest that small molecule solubilization within CDs can enhance uptake of hydrophobic agents in the brain (e.g., ~2-fold for I-GALP, a galanin-like peptide). Perhaps most significantly, different CD structures have been shown to impact regional brain distribution of small molecules in distinct ways [18,36]. Depending on the size and nature of the hydrophobic pocket, as well the surface chemistry of the CD itself, both drug equilibrium and complex transit through tissue will be altered. For instance, intranasal delivery of pituitary adenylate cyclase activating polypeptide (PACAP) to the brain showed increased uptake in several brain regions, including both the frontal cortex and the hippocampus, when solubilized within β-CD, whereas PACAP delivery via solubilization within α-CD had no significant effect in these regions [36]. The addition of α-CD for intranasal delivery of I-GALP showed the highest I-GALP uptake in the hypothalamus, followed by the olfactory bulb, and then the hippocampus, whereas the addition of methylated-β-CD showed the highest I-GALP uptake in the olfactory bulb, and relatively homogenous delivery to the other brain regions analyzed [18]. Thus, based on prior studies demonstrating CD structure-specific distribution of small molecules, and the known clinical relevance of CD-solubilized E2, we were motivated to ascertain whether different formulations of E2 would impact its brain-specific tissue distribution, and to test how CD solubilization affects function of E2 relative to control E2 solution.

The present work aimed to evaluate: (1) CD type-specific modulation of intranasal E2 brain distribution, and (2) cognitive and peripheral tissue effects of intranasal E2 in middle-aged ovariectomized rats. Study 1 evaluated brain and peripheral tissue distribution of intranasally administered, tritiated E2 solubilized freely or in one of four CD formulations. Study 2 compared the intranasal E2-CD formulation with greatest E2 uptake in cognitive brain regions versus uterine horns to intranasal free E2 solution on learning and memory and on uterine measures. We predicted that the E2-CD complex that resulted in the greatest E2 uptake in cognitively involved brain regions as compared to the uterine horns would improve spatial learning and memory and have minimal uterine stimulation relative to vehicle and relative to free E2 solution. Our results demonstrate that tissue-specific E2 bioavailability is impacted by formulation and raise important translational considerations for the use of CD to solubilize E2 for intranasal administration.

## 2. Materials and Methods

### 2.1. Materials

Carprofen (Rimadyl) was purchased from Zoetis (Parsippany, NJ, USA). Tritiated E2, dissolved in ethanol, was purchased from American Radiolabeled Chemicals (St. Louis, MO, USA). Solvable Solution and Hionic-Fluor scintillation cocktail were purchased from PerkinElmer (Waltham, MA, USA). Randomly methylated β-CD, 2HP-β-CD, β-CD, γ-CD, polyethylene glycol 300 (PEG300), heparin sodium salt, and non-radioactive E2 were purchased from Sigma-Aldrich (St. Louis, MO, USA), and ethanol was purchased from VWR (Radnor, PA, USA). Non-toxic black paint was purchased from Blick (Highland Park, IL, USA). Additional chemicals and solutions, including sodium chloride, 30% hydrogen peroxide, monobasic sodium phosphate, and dibasic sodium phosphate, were purchased from Fisher Scientific (Waltham, MA, USA).

### 2.2. Animals

**Figure 1:**
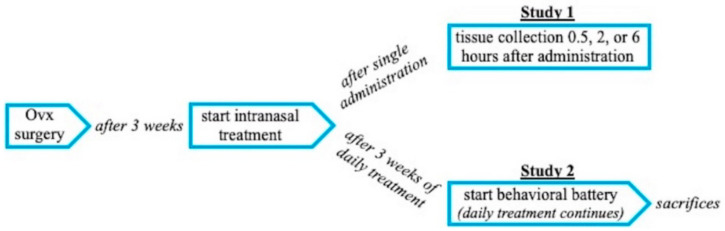
General timeline for Studies 1 and 2.

Sixty-two 9–11-month-old (Study 1) and 40 9–10-month-old (Study 2) female Fischer-344 CDF virgin rats were ordered from the National Institute on Aging, Harlan Laboratories (Indianapolis, IN, USA). All rats were pair-housed, kept on a 12-h light/dark cycle, and provided food and water ad libitum at Arizona State University. All procedures were approved by the Arizona State University IACUC (Protocol # 171580R RFC 1, Date of Action: 16 May 2017) and adhered to the standards set by the National Institutes of Health. See Figure 1 for the general study timeline.

### 2.3. Study 1: Brain-Distribution and Biodistribution of Intranasal Free E2 and E2-CD Complexes

#### 2.3.1. Ovariectomy (Ovx)

All rats received Ovx surgery under acute isoflurane anesthesia, as previously described [12,37,38]. Briefly, following dorsolateral incisions to the skin and muscle, the tip of each uterine horn was ligatured and the ovaries plus the tip of the uterine horns were excised. Muscle and skin were sutured and stapled closed, respectively. Subcutaneous carprofen (5 mg/mL/kg) was given for pain, and subcutaneous saline (2 mL) was given to prevent dehydration. One rat died due to surgical complications.

#### 2.3.2. Treatments

Approximately three weeks following Ovx surgery (20 ± 2 days after Ovx), 59 rats were randomly assigned to receive a single intranasal administration of one of five treatment formulations outlined in Table 1. For three days prior to treatment administration, all rats were habituated to intranasal administration handling. Two rats did not receive treatment and served as background radioactivity controls for later tissue processing. The five treatments were administered to each rat at 0.04 mCi of tritiated E2 per kg body weight. The formulations included free E2 solution (Treatment A), E2/randomly methylated β-CD complex (Treatment B), E2/2HP-β-CD complex (Treatment C), E2/β-CD complex (Treatment D), and E2/γ-CD complex (Treatment E). All treatments were solubilized in 0.9% saline with the exception of Treatment A, which was solubilized using 20% PEG300 solution in 0.9% saline; the pH was kept at 5.5 for all treatments to match the pH range of a rat’s nasal cavity and to minimize irritation following intranasal administration in awake rats [39]. Treatment A served as the E2 control solution, without the addition of a CD carrier. Treatment B utilized the CD that was used in the clinically available Aerodiol intranasal spray [16,30]. Treatment C utilized the CD that has been evaluated in behavioral studies of E2′s effects on learning and memory as well as neuromolecular mechanisms [33,34,35]. For formulations B and C, CDs were dissolved in 96% ethanol and combined with tritiated E2 at a 1:2 E2 to CD molar ratio. Treatment D utilized the base β-CD of the two CD derivatives from Treatments B and C; tritiated E2 was combined with the CD that was dissolved in 50% ethanol at 50 °C at a 1:4 E2 to CD molar ratio and sonicated for 30 min. Treatment E utilized γ-CD, which has a greater internal cavity size than that of β-CD [17]; tritiated E2 was combined with the CD that was dissolved in 50% ethanol at 50 °C at a 1:5 E2 to CD molar ratio and sonicated for 30 min. After the five treatment formulations were generated, each formulation was aliquoted, ethanol was evaporated, and treatments were stored at 4 °C. On the day of treatment administration, each rat was weighed, gently wrapped in a towel while exposing the nostrils, and administered 5 μL of a treatment per nostril for a total of 10 μL of tritiated E2 (0.04 mCi per kg body weight).

#### 2.3.3. Tissue Collection

At 0.5, 2, or 6 h following intranasal administration (*n* = 3−4 rats per treatment per time point), isoflurane was used to anesthetize the rat, whole blood was collected from the right atrium of the heart and frozen (−20 °C), and the rat was perfused with heparinized PBS (10 units of heparin per mL of PBS) through the left ventricle of the heart. Following perfusion, the brain was rapidly excised and dissected to collect the olfactory bulbs, trigeminal nerves, frontal cortex, cingulate cortex, basal forebrain, striatum, dorsal hippocampus, hypothalamus, amygdala, entorhinal cortex, perirhinal cortex, and the CA1/CA2 region of the ventral hippocampus. The tissue was weighed and stored at −20 °C until later processing. Uterine horns were removed from the body cavity, trimmed of visible fat, weighed, and stored at −20 °C until later processing.

#### 2.3.4. Tissue Processing

Solvable solution was used to homogenize whole blood, brain tissue, and uterine horns. For brain tissue, 0.005 mL of Solvable per mg of tissue was added to each sample and then incubated overnight at 50 °C. The next day, 0.2 mL of 30% hydrogen peroxide was added to each sample and incubated for 30 min at 50 °C for de-colorization. Hionic-Fluor scintillation cocktail was added to each sample, and radioactivity was read in triplicate 1 h later on the LS 6500 Multi-Purpose Liquid Scintillation Counter (Beckman Coulter, Brea, CA, USA). For whole blood, 0.005 mL of Solvable per mg of whole blood was added to each sample and then incubated overnight at 50 °C. The next day, to minimize foaming, 0.2 mL of 30% hydrogen peroxide was added in 50 μL increments to 500 μL of each sample and incubated for 30 min at 50 °C for de-colorization. Next, Hionic-Fluor scintillation cocktail was added to each sample and radioactivity was read in triplicate 1 h later. For uterine horns, each uterine horn was finely minced, weighed, and 0.005 mL of Solvable per mg of tissue was added to each sample and incubated overnight at 50 °C. The next day, to minimize foaming, 0.2 mL of 30% hydrogen peroxide was added in 100 μL increments to 500 μL of each sample and incubated for 30 min at 50 °C for de-colorization. Hionic-Fluor scintillation cocktail was then added to each sample and radioactivity was read in triplicate 1 h later. The tissue and whole blood from the rats that did not receive any treatment was processed in the same exact way and radioactivity was read at the same time as the respective samples were processed. All data were expressed as CPM/mg of tissue after subtracting the background CPM/mg obtained from the control rats (rats that received no treatment).

### 2.4. Study 2: Spatial Learning and Memory and Uterine Stimulation Following Daily Intranasal Free E2 and E2-CD Treatment

#### 2.4.1. Ovariectomy (Ovx)

Ovx surgery was performed on all rats following the same procedure as outlined in Study 1 methods. The first body weight measurement was collected immediately prior to Ovx and then collected weekly thereafter to monitor animal health and to confirm Ovx and E2 exposure; body weight was expected to increase following Ovx for all rats and to decrease with E2 treatment [12,40,41].

#### 2.4.2. Treatment Administration

Three weeks following Ovx surgery, all rats were randomly assigned to receive daily intranasal administration of one of four treatments that continued for the duration of the study. Treatments were counter-balanced to ensure that all rats were tested on behavioral paradigms 0.5–1.5 h following treatment administration. The four treatments followed the same synthesis and administration procedures as outlined in Study 1 methods but utilized non-radioactive E2. The four treatments included saline/PEG (20% PEG300 solution in saline) as the vehicle control for free E2, 1 μg/rat free E2, saline/2HP-β-CD as the vehicle control for the E2-CD complex, and 1 μg/rat E2/2HP-β-CD. The amount of CD in saline/2HP-β-CD and in E2/2HP-β-CD vehicle groups were matched. The dose of E2 was determined to be 1 μg/rat/day based on the lowest dose of E2 in Aerodiol that effectively alleviated symptoms of menopause in women, adjusted for difference in body weight between women and rats [16].

#### 2.4.3. Water Radial-Arm Maze (WRAM)

Spatial working and reference memory were tested with the win-shift WRAM beginning week 4 following the initiation of treatment [12,38,42,43,44]. The WRAM consisted of 8 arms (38.1 cm × 12.7 cm each) and the testing room had abundant spatial cues set up to aid in spatial navigation. The maze was filled with opaque water using non-toxic black paint, and the temperature of the water was maintained at 18–20 °C. Four out of the 8 arms contained hidden platforms that were 10 cm in diameter, the location of which was fixed throughout all days of testing for a rat but was varied across rats and within treatment groups. For each trial, there was a maximum time of 3 min allotted to solve the task by locating one of the platforms. To start a trial, a rat was dropped off at the same start arm location each time and the trial continued until the rat either found or was led to a platform (after 3 min passed). After 15 s on the platform, the rat was placed back into a heated testing cage. The inter-trial interval (ITI) was 30 s, during which the just-located platform was removed and a fishnet was used to clean the maze and redistribute olfactory cues. After the ITI, the next trial was started and the task continued until there were no more platforms left to find, resulting in four trials per day for each rat. With each trial, the task became increasingly harder to solve as working memory demand was increasingly taxed as trials progressed. After 12 days of testing, on day 13, a delay period of 6 h was added between trials 2 and 3 to evaluate delayed memory retention.

Performance on the WRAM was scored by recording and scoring the number of error arm entries. Error entries were further divided into 3 memory measures evaluating reference and working memory. A reference memory (RM) error was made when a rat entered a non-platformed arm within a day. A working memory incorrect (WMI) error was made when a rat re-entered a non-platformed arm within a day. A working memory correct (WMC) error was made when a rat entered a previously platformed arm within a day.

#### 2.4.4. Morris Water Maze (MWM)

Spatial reference memory was evaluated by the MWM starting on the day following the WRAM delay, using previously reported methods [12,45]. The maze was a circular pool (188 cm in diameter), and the testing room had abundant spatial cues set up to aid in spatial navigation. The maze was filled with 18–20 °C water made opaque with non-toxic black paint. A single platform (10 cm in diameter) was hidden beneath the surface of the water in the northeast (NE) quadrant of the maze. The platform location remained the same for all rats for 4 trials per day, and 5 days of testing. The drop-off locations (north, south, west, east) varied across trials, and the order of the drop-off locations varied each day. Once the rat was released into the water, the trial started and the rat was permitted a maximum of 60 s to find the platform, after which point they were led to the platform if it was not found. After the platform was located or the rat was led to it, the rat remained on the platform for 15 s before being placed into a heated testing cage. The ITI was approximately 5–8 min, during which the water was cleaned of any debris and olfactory cues were re-distributed. MWM performance was recorded by a camera suspended above the maze, and the EthoVision tracking system (Noldus Instruments, Wageningen, The Netherlands) was used to determine swim distance. On the 5th day of testing, a 5th trial was added as the probe trial during which the platform was removed and rats were dropped off from the west location, the furthest drop-off location from the NE quadrant, the target quadrant that previously contained the platform during all other trials. Each rat was allowed to swim for a full 60-s period, and the percentage of total distance that the rats swam in the NE quadrant relative to the opposite southwest (SW) quadrant was measured to evaluate spatial localization to the platform.

#### 2.4.5. Visible Platform

To confirm that rats were capable of performing the procedural components (e.g., visual, motor) of solving a water-escape task, the visible platform task was implemented on the day after MWM [46,47,48]. A rectangular tub (100 cm × 60 cm) was set up in a testing room with all spatial cues blocked by a curtain pulled around the maze. The maze contained 18–20 °C clear water with one black platform (10 cm in diameter) that was kept 4 cm above the water surface. The maximum trial time to find the platform was 90 s, with 6 trials total. The platform location was semi-randomly varied between trials and the ITI was 5–8 min. The trial started when a rat was dropped off from a set location and the trial ended when the rat either found the platform or was led to the platform after 90 s. Once found, each rat was given 15 s on the platform before being placed into a heated testing cage. Performance on the visible platform task was measured as time, in seconds, to platform.

#### 2.4.6. Open Field

The last behavior task, the open field, was administered the day after the visible platform task. The open field task was used to evaluate locomotor activity and anxiety-like behavior [49,50]. The apparatus was 100 × 100 × 40 cm in size and was placed in the center of a dark testing room. After at least 30 min of habituation in an adjacent testing room, each rat was brought into the testing room, placed in the apparatus at the center of the north wall, and allowed to explore the arena for 10 min. Between each rat, the open field was cleaned with water to re-distribute any olfactory cues. Rat behavior was recorded and scored using the EthoVision tracking system. Locomotor activity was measured as total distance traveled, and anxiety-like behavior was measured as time spent in the maze center (30 × 30 cm center area).

#### 2.4.7. Uterine Horn Weights

Ovarian hormones impact uterine horn weight and this can be effectively used to confirm Ovx (decrease in weight) and to assess peripheral E2 exposure (increase in weight) [12,48,51,52]. Uterine horns were excised, all visible fat was removed, and the wet weight was collected at sacrifice.

### 2.5. Statistical Analyses

For all statistical analyses, alpha was set at *p* < 0.05. Study 1 analyses included one-tailed Student’s *t*-tests to compare each E2-CD to free E2 for each time point. One outlier was excluded from all analyses and figure representations from the 0.5 h timepoint for Treatment B.

Repeated measures ANOVA was used to evaluate body weight changes before treatment, weeks 1–4, and during treatment, weeks 5–10, for Study 2. Treatment was the independent variable and Weeks were the repeated measures. One-way ANOVA was used for uterine horn weight analyses with Treatment set as the independent variable and Uterine Horn Weight set as the dependent variable. Fisher’s post hoc analyses were performed when the main of effect of Treatment was significant.

For WRAM, MWM, and visible platform tasks, a 2 × 2 factorial repeated measures ANOVA was used to evaluate performance, with Estrogen Treatment (No E2 or E2) and Vehicle Type (saline/PEG or saline/2HP-β-CD) set as factors to evaluate effects of Estrogen, Vehicle, or Estrogen × Vehicle interactions. In the case of a significant effect, additional repeated measures ANOVAs were conducted to directly evaluate Treatment effects for the appropriate two group comparison. Estrogen Treatment, Vehicle Type, or Treatment were set as the independent variables, and Trials nested within Days were the repeated measures. For the WRAM, each memory measure (WMC, WMI, and RM errors) was evaluated separately on days 2–7, the acquisition phase, and on days 8–12, the asymptotic phase, as done previously [48,49]. A significant Trial × Estrogen, Trial × Vehicle, or Trial × Estrogen × Vehicle interaction led to further analyses on the highest working memory load trial that was evaluated on this version of the WRAM, Trial 4. For the WRAM delay, total errors made (WMC, WMI, and RM memory measures combined) on the post-delay trials (Trials 3 and 4 on Day 13) were compared to the baseline trials (Trials 3 and 4 on Day 12) within each treatment group to evaluate delayed memory retention. For the MWM maze, total swim distance across the 5 days was analyzed. The probe trial was analyzed separately to assess spatial localization within each treatment group using a repeated measures ANOVA to evaluate percent swim distance in the previously platformed quadrant (northeast; NE) compared to percent swim distance in the directly opposite quadrant (southwest; SW). For the open field, 2 × 2 factorial ANOVAs were used with Estrogen Treatment and Vehicle Type set as factors and Total Distance and Time in Center as dependent variables.

## 3. Results

### 3.1. Study 1: Brain-Distribution and Biodistribution of Intranasal Free E2 and E2-CD Complexes

**Figure 2:**
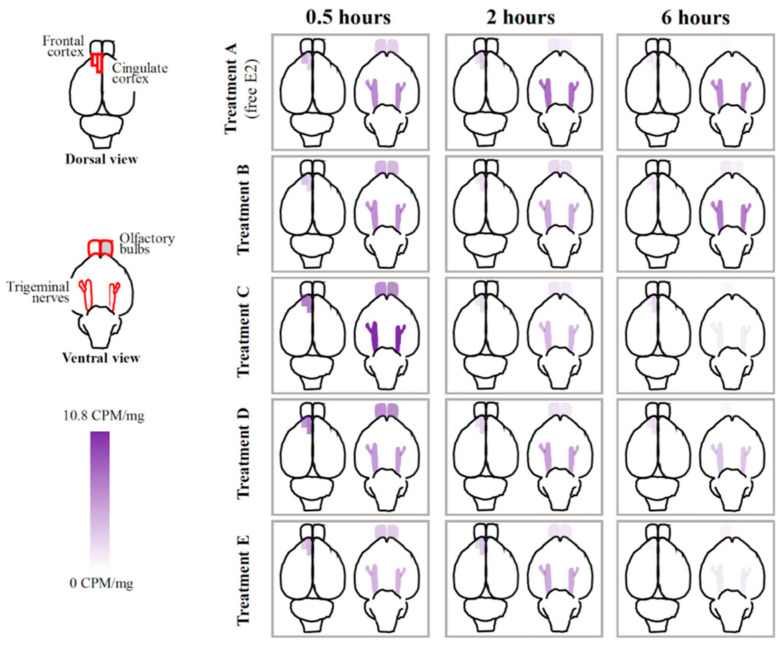
Tritiated E2 distribution in frontal cortex, cingulate cortex, olfactory bulbs, and trigeminal nerves 0.5, 2, and 6 h following intranasal administration as a function of CD type [53].

**Figure 3:**
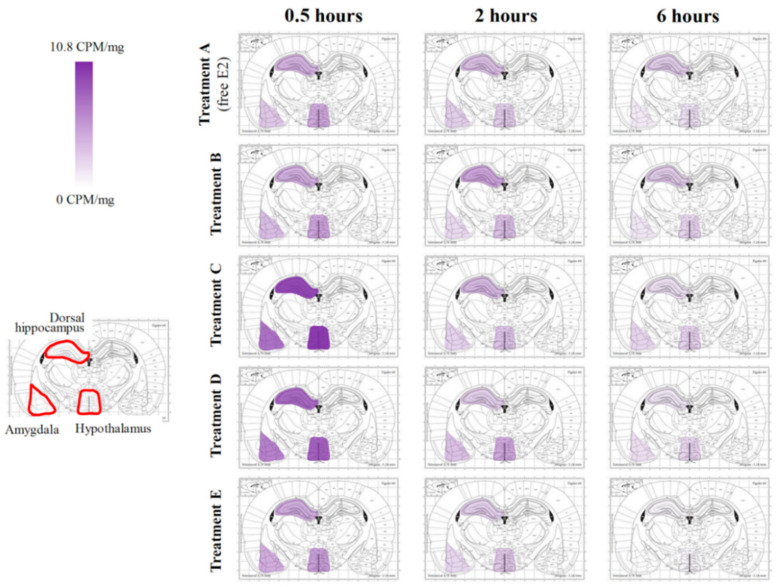
Tritiated E2 distribution in dorsal hippocampus, amygdala, and hypothalamus 0.5, 2, and 6 h following intranasal administration as a function of CD type [53].

**Figure 4:**
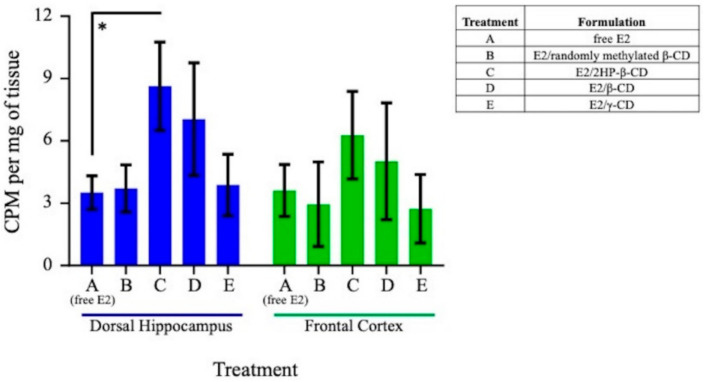
Tritiated E2 concentration in the dorsal hippocampus and frontal cortex 0.5 h following intranasal administration of Treatments A–E. All data are represented as mean ± s.e.m. * *p* < 0.05.

**Figure 5:**
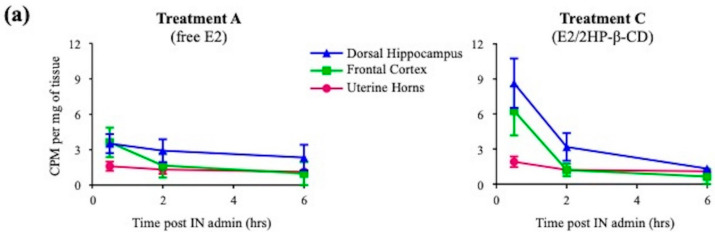

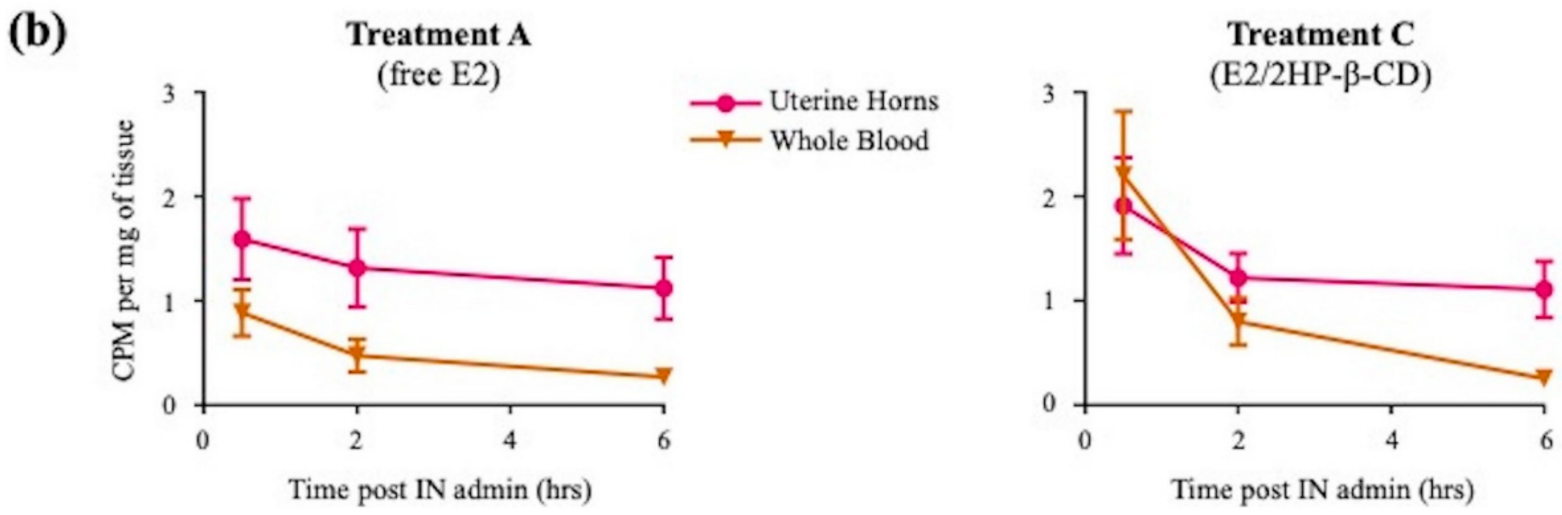
Tritiated E2 concentration in dorsal hippocampus, frontal cortex, uterine horns, and whole blood across time after intranasal administration of Treatment A and Treatment C. (**a**) Tritiated E2 concentration in dorsal hippocampus, frontal cortex, and uterine horns 0.5, 2, and 6 h following intranasal administration of Treatment A and Treatment C; (**b**) tritiated E2 concentration in uterine horns and whole blood 0.5, 2, and 6 h following intranasal administration of Treatment A and Treatment C. All data are represented as mean ± s.e.m.

Delivery of E2 varied across both brain regions and time as a function of formulation. Representative spatial heat maps were created to illustrate the distribution of E2 as a function of CD type (Figure 2 and Figure 3, Supplementary Figures S1 and S2). The evaluated brain regions included olfactory bulbs, trigeminal nerves, frontal cortex, cingulate cortex, basal forebrain, striatum, dorsal hippocampus, hypothalamus, amygdala, entorhinal cortex, perirhinal cortex, and the CA1/CA2 region of the ventral hippocampus. In general, the greatest E2 delivery to each brain area was observed at the earliest time point collected, 0.5 h, followed by a rapid decrease for each treatment formulation. E2 concentration in the trigeminal nerves, one of the potential pathways for agent uptake into the brain with intranasal delivery [20], tended to have slower clearance across the three evaluated time points as compared to the olfactory bulbs, another potential pathway for agent uptake into the brain with intranasal delivery (Figure 2). Focusing on the two regions of interest, E2 in the dorsal hippocampus 0.5 h following Treatment C administration was significantly greater than that of Treatment A (*p* < 0.05; Figure 4). Furthermore, when comparing the dorsal hippocampus to the uterine horns after E2 delivery, Treatment A resulted in ~2-fold increased E2 levels in dorsal hippocampus relative to uterine horns, whereas Treatment C resulted in ~4-fold increased E2 levels in dorsal hippocampus relative to uterine horns (Figure 5a). Interestingly, E2 levels in the dorsal hippocampus following Treatment A (free E2) did not exhibit rapid clearance across the collected time points as seen with Treatment C. No statistically significant differences in E2 delivery to the dorsal hippocampus were observed with the other three Treatment formulations (Figure 4), and at the other collected time points for all Treatment formulations, relative to free E2. There were no statistically significant differences in E2 delivery to the frontal cortex with any E2-CD Treatment formulations relative to free E2 (Figure 4).

E2 levels in whole blood were greatest at the 0.5 h time point following intranasal administration for each Treatment group and cleared rapidly across time (Figure 5b). Uterine horn E2 levels were also greatest at the 0.5 h time point following intranasal administration for each Treatment group and cleared across time (Figure 5b). Additionally, uterine horn E2 levels were in general greater than whole blood E2 levels, with greatest difference at the 6 h time point (Figure 5b). These findings are consistent with prior biodistribution studies evaluating the fate of exogenously administered tritiated E2, where E2 appeared to be retained for longer periods of time in estrogen-responsive tissues [54,55,56].

### 3.2. Study 2: Spatial Learning and Memory, and Uterine Stimulation, Following Daily Intranasal Free E2 and E2-CD Treatment

#### 3.2.1. Body Weight

**Figure 6:**
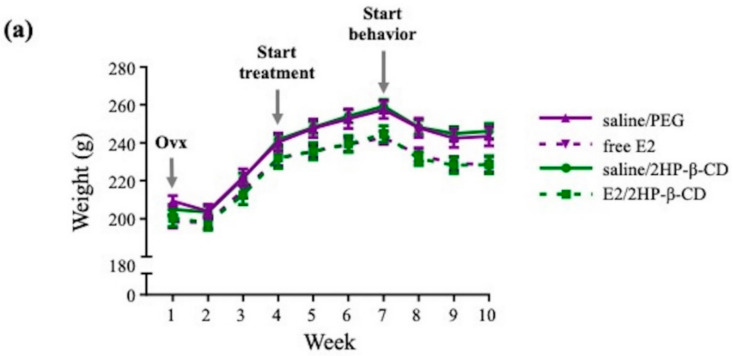

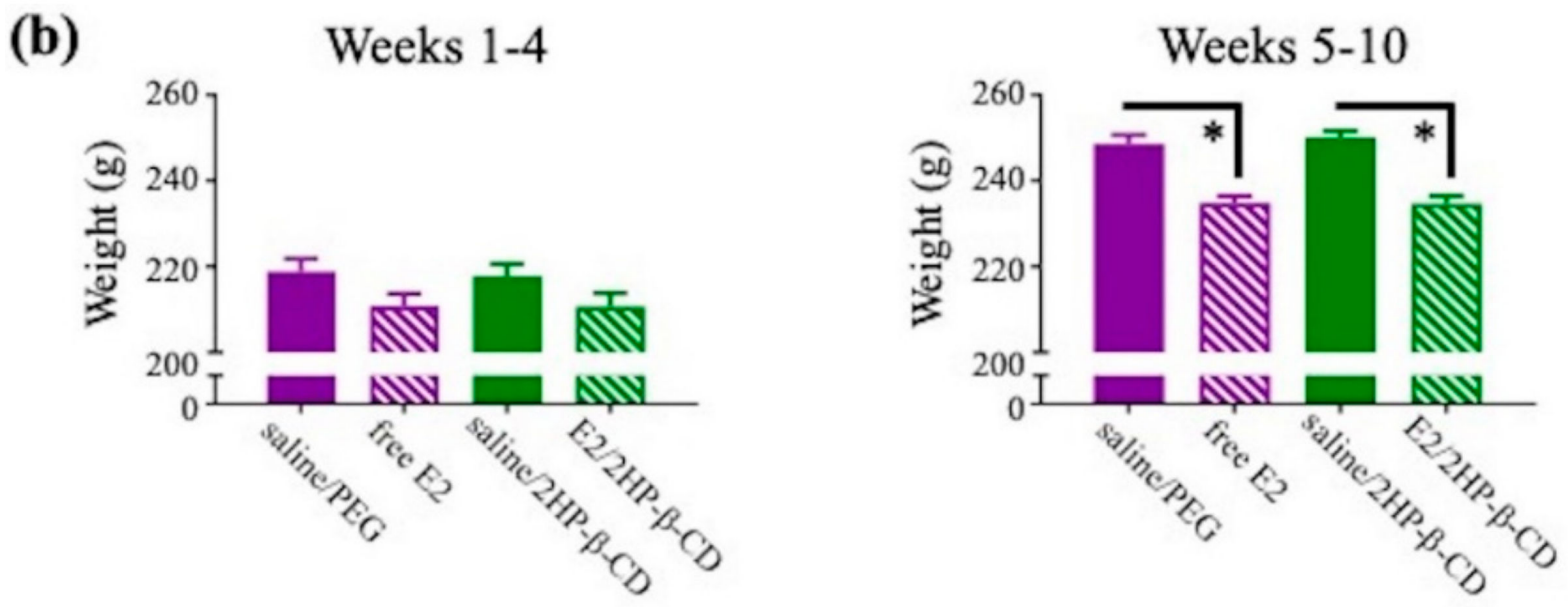
Rat body weight throughout the duration of Study 2. (**a**) Weekly body weight across the study, depicting changes in rat weight with Ovx, treatment initiation, and behavior initiation; (**b**) treatment effects on body weight in the weeks before treatment initiation, weeks 1–4, and the weeks after treatment initiation, weeks 5–10. All data are represented as mean ± s.e.m. * *p* < 0.05.

Average body weights were monitored for the duration of the study across each treatment group as a marker of health and as a general marker of E2 presence (Figure 6a) [12]. Across the first 4 weeks of the study, prior to the initiation of daily intranasal treatment, there was no main effect of Treatment for average body weight (Figure 6b). For the latter 6 weeks of the study, when daily intranasal treatment was administered, there was a main effect of Treatment for average body weight (F_(3,36)_ = 4.276, *p* < 0.05; Figure 6b), with free E2 and E2/2HP-β-CD exhibiting significantly lower body weights relative to their respective vehicle controls (*p* < 0.05), confirming the presence of E2. Body weight between free E2 and E2/2HP-β-CD treatment groups was not significantly different.

#### 3.2.2. Water Radial-Arm Maze (WRAM)

**Figure 7:**
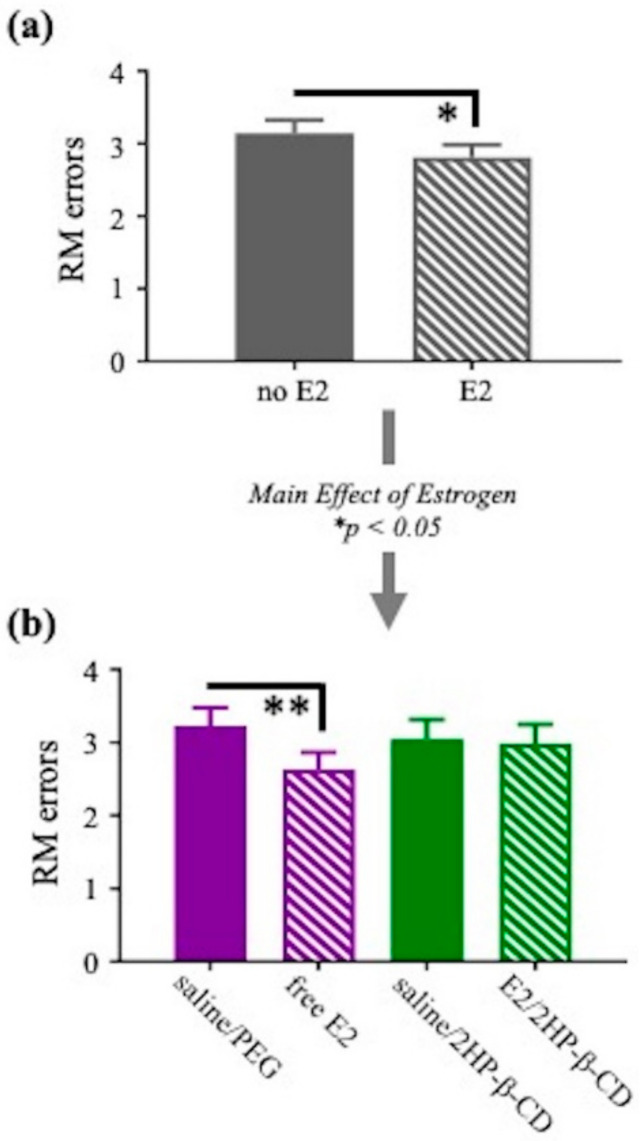
Reference memory (RM) errors made on the acquisition phase (days 2–7) of the WRAM. (**a**) Main effect of Estrogen on RM errors; (**b**) two group comparisons for further interpretation of the main effect of Estrogen on RM performance. All data are represented as mean ± s.e.m. * *p* < 0.05, ** *p* < 0.01.

Spatial working and spatial reference memory were evaluated by the WRAM. The 12 days of testing were divided into the acquisition phase, days 2–7, when the rats were learning the rules of the task, and the asymptotic phase, days 8–12, after the rats should have learned the rules of the task. During the acquisition phase, there were no main effects of Estrogen, Vehicle, or Estrogen × Vehicle Interactions for WMC errors and WMI errors. For RM errors, there was a main effect of Estrogen (F_(1,36)_ = 5.161, *p* < 0.05; Figure 7a) and a marginal Estrogen × Vehicle interaction (F_(1,36)_ = 3.303, *p* < 0.1), whereby E2-treated groups made fewer RM errors than vehicle-treated groups. Further analyses revealed that these effects were driven by a main effect of Treatment between saline/PEG and free E2 (F_(1,18)_ = 8.452, *p* < 0.01; Figure 7b), where the free E2 group made fewer RM errors than the saline/PEG group. Additionally, there was a marginal Treatment effect between free E2 and E2/2HP-β-CD (F_(1,18)_ = 3.349, *p* < 0.1), where the free E2 group tended to make fewer RM errors than the E2/2HP-β-CD group. There were no Trial × Estrogen, Trial × Vehicle, or Trial × Estrogen × Vehicle interactions for WMC, WMI, or RM errors. Together, these data indicate that the free E2 treatment improved reference memory on the acquisition phase of the WRAM.

**Figure 8:**
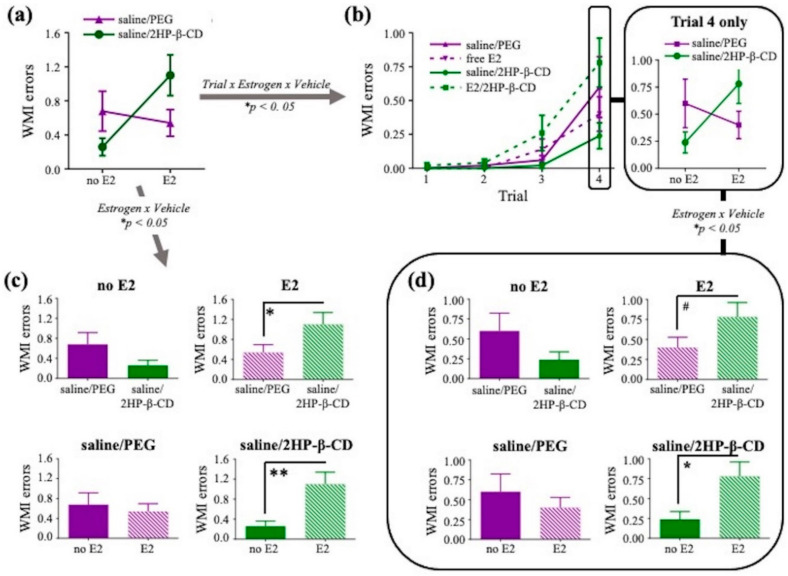
Working memory incorrect (WMI) errors made on the asymptotic phase (days 8–12) of the WRAM. (**a**) The Estrogen × Vehicle interaction for WMI errors; (**b**) the Trial × Estrogen × Vehicle interaction for WMI errors as well as the Estrogen × Vehicle interaction for Trial 4 only, the highest working memory load trial; (**c**) two group comparisons for further interpretation of the Estrogen × Vehicle interaction; (**d**) two group comparisons for further interpretation of the Trial × Estrogen × Vehicle interaction. All data are represented as mean ± s.e.m. * *p* < 0.05, ** *p* < 0.01, ^#^ *p* < 0.1.

During the asymptotic phase, there were no main effects of Estrogen or Vehicle, or interactions of Estrogen × Vehicle, Trial × Estrogen, Trial × Vehicle, or Trial × Estrogen × Vehicle, for WMC errors and RM errors. For WMI errors, there was a significant Estrogen × Vehicle interaction (F_(1,36)_ = 5.414, *p* < 0.05; Figure 8a) and a significant Trial × Estrogen × Vehicle interaction (F_(3,108)_ = 3.522, *p* < 0.05; Figure 8b). Further analyses for WMI errors, collapsed across all four trials, revealed a main effect of Treatment between E2/2HP-β-CD and saline/2HP-β-CD (F_(1,18)_ = 9.885, *p* < 0.01; Figure 8c) and between E2/2HP-β-CD and free E2 (F_(1,18)_ = 4.449, *p* < 0.05; Figure 8c), where the E2/2HP-β-CD group made more WMI errors compared to the saline/2HP-β-CD group and compared to the free E2 group. Additionally, in Trial 4, the highest working memory load trial evaluated on the WRAM in the present study, there was a significant Estrogen × Vehicle interaction for WMI errors (F_(1,36)_ = 4.766, *p* < 0.05; Figure 8b). Further analyses for WMI errors made in Trial 4 alone revealed a main effect of Treatment between E2/2HP-β-CD and saline/2HP-β-CD (F_(1,18)_ = 6.047, *p* < 0.05; Figure 8d) and a marginal effect of Treatment between E2/2HP-β-CD and free E2 (F_(1,18)_ = 3.157, *p* < 0.1; Figure 8d), whereby the E2/2HP-β-CD group made more WMI errors than the saline/2HP-β-CD group and tended to make more WMI errors than the free E2 group. Together, these data indicate that the E2/2HP-β-CD treatment impaired working memory on the asymptotic phase of the WRAM, particularly at the highest working memory demand.

For the WRAM delay, there was no difference for total errors made (WMC, WMI, and RM memory measures combined) on the post-delay trials, Trials 3 and 4 on Day 13, compared to the baseline trials, Trials 3 and 4 on Day 12, for each treatment group, suggesting that no treatment group exhibited delay-induced forgetting following a 6-h delay period.

#### 3.2.3. Morris Water Maze (MWM)

Spatial reference memory was examined on the MWM. There were no significant effects of Estrogen, Vehicle, or an Estrogen × Vehicle interaction for swim distance to the platform across the 5 days of testing. There was a significant Day × Vehicle interaction effect (F_(4,144)_ = 2.465, *p* < 0.05) but no Day × Estrogen or Day × Estrogen × Vehicle interaction effects, suggesting that there was a difference in learning between the vehicle used for delivery regardless of E2 presence. Specifically, saline/PEG groups swam longer distance to platform on Day 2 (F_(1,38)_ = 5.507, *p* < 0.05) and tended to swim longer distance to platform on Day 3 (F_(1,38)_ = 3.797, *p* = 0.06) of MWM compared to saline/2HP-β-CD groups (Supplementary Figure S3a). A main effect of Quadrant was seen for all group comparisons for the probe trial, where percent swim distance was greater in the target NE quadrant compared to the opposite SW quadrant for saline/PEG (F_(1,9)_ = 189.448, *p* < 0.0001), free E2 (F_(1,9)_ = 99.826, *p* < 0.0001), saline/2HP-β-CD (F_(1,9)_ = 162.971, *p* < 0.0001), and E2/2HP-β-CD (F_(1,9)_ = 110.802, *p* < 0.0001) treatment groups. Together, these results indicate that all treatment groups were able to learn and spatially localize to the platform location by the end of testing, with an impact of the vehicle used for E2 delivery on learning trajectory.

#### 3.2.4. Visible Platform Task

The visible platform task assessed whether subjects across all groups possessed the motor and visual abilities to complete a water-escape task. There were no main effects of Estrogen or Vehicle, or an Estrogen × Vehicle interaction. There was a main effect of Trial (F_(5, 180)_ = 7.770, *p* < 0.0001), and no Trial × Estrogen, Trial × Vehicle, or Trial × Estrogen × Vehicle interactions, suggesting that all groups had a similar procedural capability to learn and complete a water-escape task (Supplemental Figure S3b). Each treatment group’s average latency to platform across all 6 trials was under 9 s.

#### 3.2.5. Open Field

Locomotor activity and anxiety-like behavior were assessed in the open field task. There were no main effects of Estrogen or Vehicle, or an Estrogen × Vehicle interaction, for time spent in the center, suggesting similar anxiety-like profiles across treatment groups (Supplemental Figure S3c). Although there were no main effects of Estrogen or Vehicle for total distance traveled, there was a significant Estrogen × Vehicle interaction (F_(1, 36)_ = 6.315, *p* < 0.05; Supplemental Figure S3d). Further analyses for total distance traveled revealed a main effect of Treatment between saline/2HP-β-CD and saline/PEG (F_(1,18)_ = 4.85, *p* < 0.05), with the saline/PEG group traveling a greater total distance compared to the saline/2HP-β-CD group. Additionally, there were marginal effects of Treatment between E2/2HP-β-CD and saline/2HP-β-CD (F_(1,18)_ = 3.141, *p* = 0.09) and between free E2 and saline/PEG (F_(1,18)_ = 3.141, *p* = 0.09), with the E2/2HP-β-CD group tending to travel a greater total distance than its vehicle control, and the free E2 group tending to travel less distance than its vehicle control. Together, these data suggest that the type of vehicle used for intranasal E2 delivery differentially impacts locomotor activity, an effect that interacts with E2 presence.

#### 3.2.6. Uterine Horn Weight

**Figure 9:**
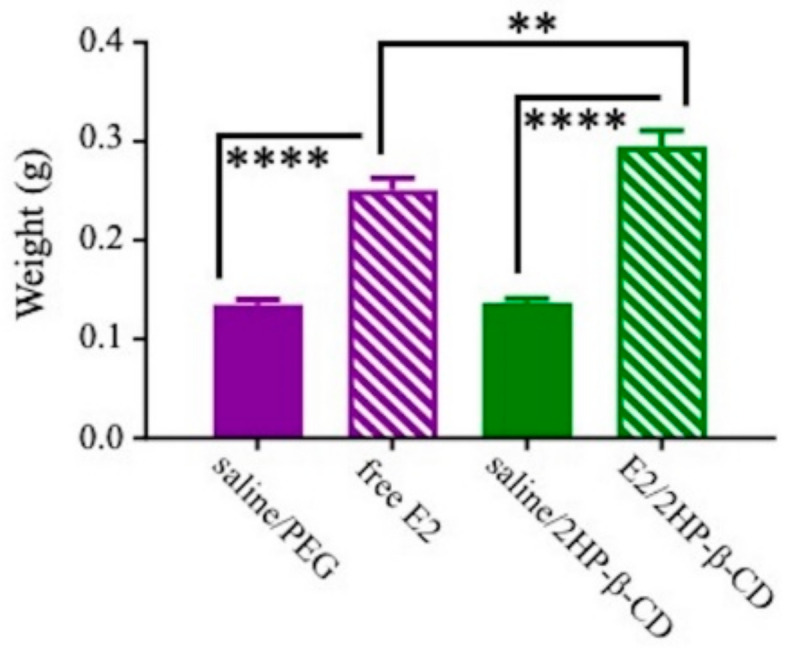
Uterine horn weights across treatment groups. All data are represented as mean ± s.e.m. ** *p* < 0.01, **** *p* < 0.0001.

To evaluate the uterine effects of the E2 regimens tested here, uterine horn weight was examined across treatments. There was a main effect of Treatment (F_(3,36)_ = 58.043, *p* < 0.0001; Figure 9), where both of the E2-treated groups had significantly higher uterine horn weights than their respective vehicle control groups (*p* < 0.0001). Additionally, the E2/2HP-β-CD group had greater uterine horn weight relative to the free E2 group (*p* < 0.01), suggesting uterine-stimulating effects with both E2 treatments, and that these effects were more pronounced with intranasal E2/2HP-β-CD treatment relative to free E2.

## 4. Discussion

The present work evaluated spatial distribution, cognitive impact, and uterine effects of free E2 and an E2-CD complex administered by the intranasal route in middle-aged, Ovx rats. Study 1 compared the spatial distribution of tritiated E2 as a function of CD type in the brain and in the periphery in relation to free E2. Study 2 examined cognitive and uterine effects of daily intranasal treatment of free E2 and of the E2-CD complex that resulted in greatest E2 uptake in the dorsal hippocampus, E2/2HP-β-CD. Daily intranasal administration of free E2 improved spatial reference memory, whereas daily intranasal administration of E2/2HP-β-CD, which was shown to exhibit greater peak E2 delivery to the dorsal hippocampus than free E2, impaired spatial working memory. Furthermore, although both E2-containing treatment groups, free E2 and E2/2HP-β-CD, increased uterine horn weight relative to their respective vehicle control groups, the E2/2HP-β-CD complex resulted in greater uterine horn weight than free E2, suggesting increased uterine stimulation by E2 with 2HP-β-CD as the delivery vehicle.

Although Study 1 evaluated E2 delivery across multiple regions in the brain, the primary focus was on E2 delivery across time to the frontal cortex and to the dorsal hippocampus as a function of CD type, as these regions are impacted by E2 and are associated with the cognitively-beneficial effects of E2 [12,14,15,57]. E2 delivery to the frontal cortex and the dorsal hippocampus peaked at the earliest time point evaluated, half an hour following intranasal administration, for all treatment formulations. Peak E2 delivery to the dorsal hippocampus was greater when solubilized with 2HP-β-CD compared to freely solubilized E2, but not when solubilized with randomly methylated β-CD complex, β-CD, or γ-CD. Furthermore, E2 was rapidly cleared from the dorsal hippocampus when administered with 2HP-β-CD, whereas E2 exhibited a more sustained presence in the dorsal hippocampus over time when administered freely. The four CD formulations did not significantly alter E2 peak delivery in the frontal cortex compared to free E2. Together, these findings suggest that the addition and type of CD can differentially impact region-specific brain uptake of E2 following intranasal administration. In relation to alternate small hydrophobic molecules, intranasal administration of PACAP solubilized with β-CD, but not α-CD, increased uptake of PACAP in several brain regions, including the frontal cortex and hippocampus [36]. In contrast, although α-CD and methylated-β-CD both increased I-GALP uptake in the brain following intranasal administration, I-GALP uptake was highest in the hypothalamus, followed by olfactory bulb, and then hippocampus when solubilized with α-CD [18]. I-GALP uptake was highest in olfactory bulb with relatively homogenous delivery to other brain regions when solubilized with methylated-β-CD for intranasal delivery [18]. When considering findings from the present study in concert with this prior work, it appears that CD type and the hydrophobic molecule interact to modulate brain region-specific uptake of the molecule following intranasal administration.

The overarching goal of intranasal E2 administration was to take advantage of the direct nose-to-brain pathway as a function of solubilizing vehicle to achieve greater E2 delivery to cognitively-involved brain regions in relation to uterine tissue. Following intranasal administration of free E2 and of E2/2HP-β-CD complex, E2 levels in whole blood and in the uterine horns were greatest at the half an hour time point. E2 levels in whole blood were rapidly cleared, whereas E2 levels in uterine horns appeared to be retained and not cleared as rapidly across the collected time points. These results were not surprising and are consistent with pharmacokinetic profiles of intravenous and subcutaneous tritiated E2 administration, whereby E2 is retained for longer periods of time in estrogen-responsive tissues, specifically the uterine horns, in relation to circulating levels [54,55,56]. Furthermore, administration of free E2 resulted in ~2-fold greater E2 levels in the dorsal hippocampus, and E2/2HP-β-CD complex resulted in ~4-fold greater E2 levels in the dorsal hippocampus, in relation to the uterine horns half an hour following intranasal administration. Thus, greater brain to peripheral tissue E2 delivery was evidenced with the intranasal route of administration, which was further enhanced with the addition of 2HP-β-CD as a solubilizing agent.

Study 2 evaluated spatial working and reference memory simultaneously on the WRAM. When rats were learning the rules of the task, during the acquisition phase, daily free E2 treatment improved reference memory performance relative to vehicle treatment. The daily free E2 treatment group also tended to make fewer RM errors than the E2-CD complex group (E2/2HP-β-CD treatment) during the acquisition phase. On the WRAM, the RM measure is capped at a maximum of four possible errors that can be made within a day, as four out of the eight available arms never contain a platform, and a RM error entry is defined as a first entry into a non-platformed arm. Therefore, even with the inherent ceiling effect, the impact of the free E2 daily intranasal treatment on the reference memory measure was robust enough to reveal a significant beneficial effect. This cognitively-beneficial effect of intranasal free E2 treatment aligns well with improved spatial reference memory effects of subcutaneous and oral E2 as evaluated on the MWM [8,10,58,59,60,61,62]. During the asymptotic phase, when rules of the WRAM should have been learned, and subjects have reached asymptotic performance, an estrogen by vehicle interaction was observed. Specifically, the E2/2HP-β-CD complex treatment impaired spatial working memory compared to its vehicle control and compared to free E2, an effect that was also present when working memory load was most taxed—on the highest working memory load trial of the task. Taken together, the WRAM findings fail to support the study hypothesis in which we predicted that greater E2 peak delivery to the dorsal hippocampus would result in enhanced cognitively-beneficial effects of E2. One possible explanation for this is that the E2 concentrations achieved in the dorsal hippocampus with daily E2/2HP-β-CD complex treatment may be too high to yield beneficial effects of E2 on spatial learning and memory. Indeed, a study in naturally cycling women found that when E2 levels were supraphysiological, the effects of E2 on hippocampal activity exhibited an inverted-U shape, suggesting that a specific range for E2 levels in the hippocampus yields a desired outcome, at least in terms of hippocampal activity, and E2 levels lower or higher than that range may in fact yield the opposite outcome [63]. In support of this notion, rodent studies evaluating different doses of subcutaneous E2 treatment have shown cognitive impairments with higher doses of E2 and cognitive improvements with lower doses of E2 [8,64]. For instance, acute subcutaneous 0.3 μg E2/rat treatment that yields physiologically low circulating E2 levels improved, whereas acute subcutaneous E2 treatment that yields physiologically high (1 μg E2/rat) or supraphysiological (10 μg E2/rat) circulating E2 levels impaired, hippocampus-sensitive learning and memory [64]. An alternate consideration for our observed cognitive results is that total E2 exposure in cognitive brain regions could be more important than peak levels, as E2/2HP-β-CD appeared to be cleared more rapidly from the dorsal hippocampus, whereas free E2 exhibited a more sustained concentration over time. Although cyclic and tonic E2 regimens at certain doses can improve spatial learning and memory in Ovx rodents, several studies have found that a more tonic E2 treatment regimen representative of greater total and constant E2 exposure, such as through a constantly releasing subcutaneous E2 pellet, results in improved spatial learning and memory outcomes as opposed to more cyclic E2 treatment regimens representative of repeated peak E2 exposures, such as weekly or every other week injections [8,12,50,65,66]. Therefore, dose and frequency of E2 treatment regimen impacts accompanying E2 exposure in the brain, which matters for directionality of spatial learning and memory outcomes. Results from the present study provide a baseline for the evaluation of intranasally administered E2 on spatial learning and memory; additional investigation of how specific treatment regimens (e.g., dose and frequency of treatment) impact cognitive effects of intranasal E2 or E2-CD is warranted.

On the MWM, spatial reference memory did not differ between treatment groups as all groups were able to learn and spatially localize to the platform location by the end of testing. Interestingly, a day by vehicle interaction revealed an impact of the vehicle used for intranasal E2 solubilization and delivery on learning trajectory. Specifically, saline/PEG groups swam a longer distance to the platform on Day 2 and tended to swim a longer distance to the platform on Day 3 compared to saline/2HP-β-CD groups, regardless of E2 presence. These findings contrast the beneficial effects of free E2 during the acquisition phase of the WRAM, which may be at least in part explained by the prior cognitive experience of the WRAM before MWM testing. Indeed, behavioral studies suggest that prior learning experience can modulate subsequent learning and memory performance [67,68]. In addition, the beneficial effects of daily free E2 treatment on reference memory on the WRAM, but not the MWM, may be due, in part, to the added burden of working memory in the WRAM task, as both working and reference memory are tested simultaneously thereby adding to overall memory demand. Importantly, the visible platform task that followed MWM testing revealed that all treatment groups were able, and exhibited similar abilities, to complete a water-escape task. These findings demonstrate that the treatment paradigms within the study were not detrimental to performance on the procedural components of a water-escape task, and that groups did not differ within this context. Additionally, no treatment differences were observed on the anxiety-like behavior measure of the open field task. There was, however, an estrogen by vehicle interaction for locomotor activity on the open field task indicating a vehicle effect, with the saline/PEG group exhibiting greater locomotor activity compared to the saline/2HP-β-CD group, an effect that was negated by the presence of E2. The two vehicles, PEG300 and 2HP-β-CD, have been successfully employed as solubilizing agents for E2 treatment in previous behavioral studies. Indeed, E2 improves learning and memory when solubilized in PEG300 for subcutaneous release from Alzet pumps as well as when solubilized in 2HP-β-CD for acute intraperitoneal injections [69,70]. However, the distinct contribution of each vehicle on the effect of intranasal E2 across multiple behavioral outcomes has not been previously evaluated. Present findings suggest that the vehicle used for intranasal E2 delivery can impact behavioral outcomes and underscore the importance of selecting an appropriate vehicle for E2 treatment administration within a behavioral study design.

A complete Ovx procedure and the presence of E2 in Study 2 were confirmed via body and uterine horn weight measurements. Following Ovx, all treatment groups exhibited increases in average body weights that did not differ from each other, confirming successful Ovx, as rodents tend to gain weight rapidly following the removal of both ovaries [12,40]. After daily intranasal treatment initiation, which continued for the rest of the study, both of the E2-containing treatments continued to exhibit weight gain but at a lower rate than the two vehicle control groups, resulting in significantly lower average body weight with E2-containing treatments compared to their respective vehicle controls. There was no difference in body weight between free E2 and E2/2HP-β-CD complex treatments. This E2-induced divergence in body weight between free E2 and its vehicle control, and E2/2HP-β-CD complex and its vehicle control, was expected based on prior work and confirmed the presence of E2 following intranasal administration [12]. Importantly, change in body weight is not a direct marker of peripheral E2 exposure, as studies have shown that estrogen receptors in the hypothalamus can mediate the observed changes in body weight [71]. A more direct measure of peripheral exposure to E2 are uterine horn weights, which are expected to decrease with Ovx and increase with E2 exposure [12,48,51,52]. This was confirmed in the present study, as uterine horn weight was greater with free E2 and E2/2HP-β-CD complex treatments relative to their respective vehicle controls, verifying Ovx as well as E2 presence at the uterine tissue. Interestingly, uterine horn weights were greater with E2/2HP-β-CD complex treatment relative to free E2 treatment, suggesting potentially greater or longer peripheral exposure of the uterine tissue to E2 when the estrogen was complexed with the 2HP-β-CD compared to without. Together, the uterine horn weight results indicate that the cognitively-beneficial daily free E2 intranasal treatment had lower uterine stimulation relative to the cognitively-impairing daily E2/2HP-β-CD complex treatment.

## 5. Conclusions

In conclusion, daily intranasal administration of the E2-CD complex that yielded the greatest peak E2 uptake in the dorsal hippocampus impaired spatial working memory and also had the greatest undesired effect on uterine stimulation in middle-aged, Ovx rats. However, daily intranasal free E2 treatment improved spatial reference memory and yielded lower uterine stimulation than E2-CD complex treatment. In the clinic, intranasal E2-CD has been shown to be effective at reducing common symptoms of menopause (e.g., hot flashes); however, its effects on cognition were not assessed prior to its commercial discontinuation [16,72]. The data in the present study suggest that intranasal E2 therapy should be re-visited for clinical effects, particularly focusing on its potential therapeutic effects on cognition in concert with treatment for other common symptoms of menopause (e.g., hot flashes) as a function of formulation considerations such as pharmacokinetic profiles in specific target tissues. Future work can focus on alternate E2 carriers, such as chitosan or gelatin nanoparticles, for increased E2 nasal absorption and delivery to the brain [73,74]. Importantly, the cognitively-beneficial effects of intranasal E2 provide novel avenues for further assessment of clinically-available estrogen plus progestogen combinations for cognitive therapy; indeed, the cognitive and uterine effects of combination hormone treatments where the estrogen is administered intranasally and the progestogen is administered either subcutaneously or locally (i.e., intrauterine devices) are currently unexplored. Combining and taking advantage of the inherent pharmacokinetic properties of different routes of administration and/or carrier types may yield the optimal delivery platform for menopausal hormone therapy, whereby the beneficial cognitive effects of estrogens are maximized and the undesired uterine stimulation effects are minimized. Continuing the evaluation of the cognitive, brain localization, and peripheral effects of intranasal E2 treatment, and the varying factors surrounding it, is critical as the information gained can lead to developments in hormone therapy formulations targeting particular symptoms and risk factors in menopausal women.

## Figures and Tables

**Table 1:** Summary of treatment groups for Study 1.

Treatment	Formulation	E2:CD Molar Ratio	Aqueous Medium	Rationale for CD
A	free E2	-	20% PEG300 saline	-
B	E2/randomly methylated β-CD	1:2	Saline	CD derivative found in Aerodiol intranasal spray
C	E2/2HP-β-CD	1:2	Saline	CD derivative used in behavioral studies evaluating estrogen effects on the brain
D	E2/β-CD	1:4	Saline	Base CD for the derivatives used in treatments B and C
E	E2/γ-CD	1:5	Saline	CD with a larger inner cavity than β-CD

## Supplementary Materials

The following are available online at https://www.mdpi.com/1999-4923/12/12/1225/s1, Figure S1: Tritiated E2 distribution in striatum and basal forebrain 0.5, 2, and 6 h following intranasal administration as a function of CD type [53]. Figure S2: Tritiated E2 distribution in ventral CA1/CA2 hippocampus, entorhinal cortex, and perirhinal cortex 0.5, 2, and 6 h following intranasal administration as a function of CD type [53]. Figure S3: Performance on the MWM, visible platform task, and open field task. (**a**) Total distance traveled to platform across days on the MWM; (**b**) time to platform across trials on the visible platform task; (**c**) time spent in the center of the open field task; (**d**) Estrogen × Vehicle interaction for total distance traveled on the open field task. All data are represented as mean ± s.e.m. * *p* < 0.05.
