# Supplementary material for: Intranasal 17β-Estradiol Modulates Spatial Learning and Memory in a Rat Model of Surgical Menopause
Source: Pharmaceutics. 2020 Dec 17;12(12):1225. doi: 10.3390/pharmaceutics12121225 (PMC7766209; doi:10.3390/pharmaceutics12121225)
Supplement: Supplementary file 1 [file pharmaceutics-12-01225-s001.pdf]

pharmaceutics

Intranasal 17β-Estradiol Modulates Spatial Learning and Memory in a Rat Model of Surgical Menopause

Alesia V. Prakapenka, Veronica L. Peña, Isabel Strouse, Steven Northup-Smith, Ally Schrier, Kinza Ahmed, Heather A. Bimonte-Nelson and Rachael W. Sirianni *

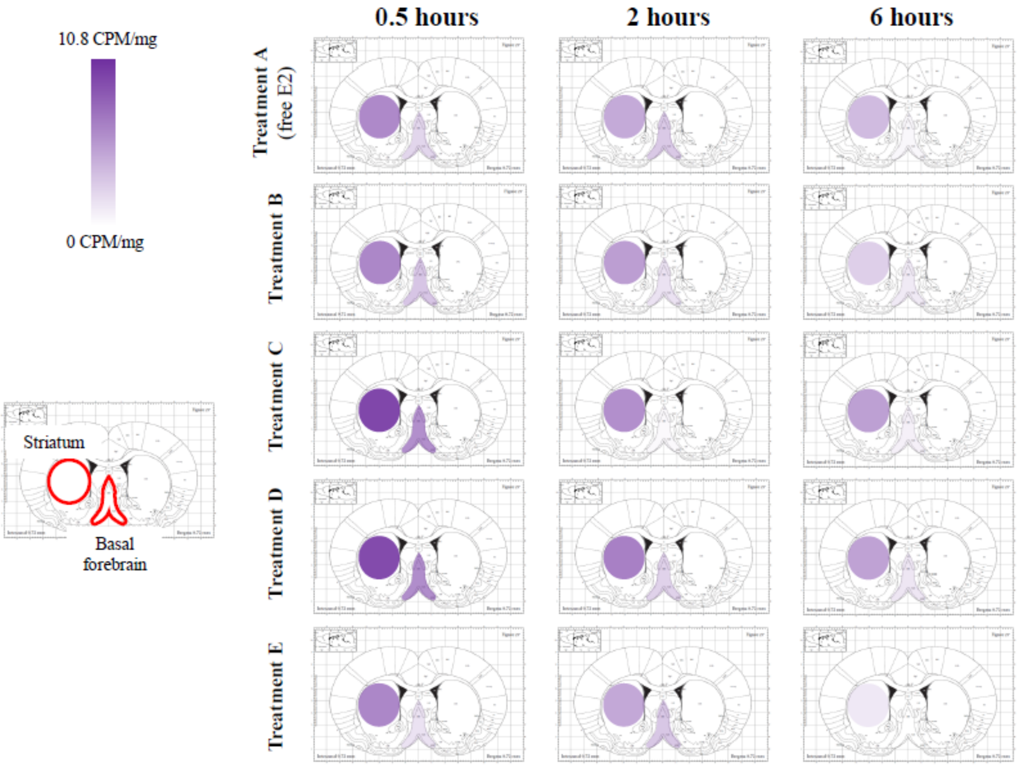

Figure S1. Tritiated E2 distribution in striatum and basal forebrain 0.5, 2, and 6 h following intranasal administration as a function of cyclodextrin type [54].

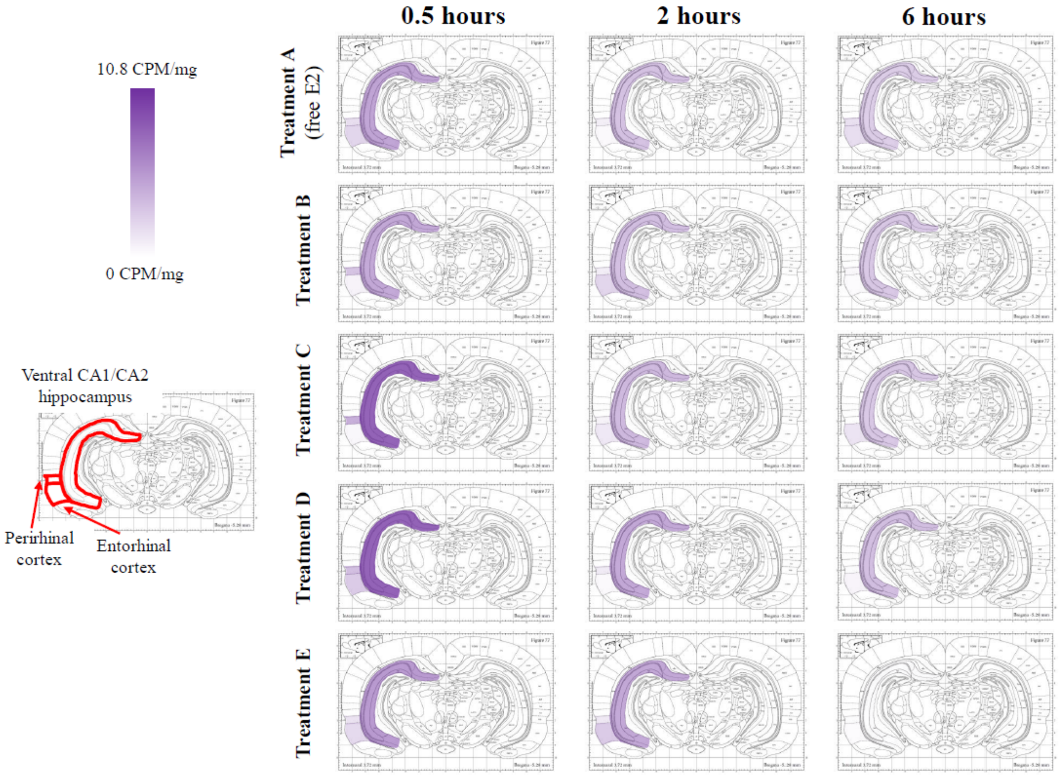

Figure S2. Tritiated E2 distribution in ventral CA1/CA2 hippocampus, entorhinal cortex, and perirhinal cortex 0.5, 2, and 6 h following intranasal administration as a function of cyclodextrin type [54].

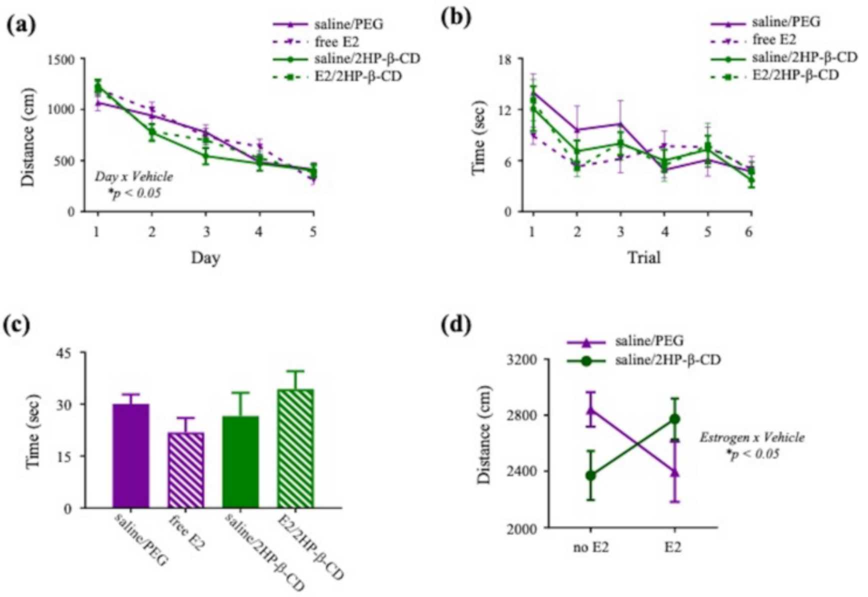

Figure S3. Performance on the MWM, visible platform task, and open field task. (**a**) Total distance traveled to platform across days on the MWM; (**b**) time to platform across trials on the visible platform task; (**c**) time spent in the center of the open field task; (**d**) Estrogen × Vehicle interaction for total distance traveled on the open field task. All data are represented as mean ± s.e.m. * $p < 0.05$.
